# Prevalence, risk factors, and prediction of inappropriate use of non-vitamin K antagonist oral anticoagulants in elderly Chinese patients with atrial fibrillation: A study protocol

**DOI:** 10.3389/fcvm.2022.951695

**Published:** 2022-08-24

**Authors:** Shu-Juan Zhao, Bo-Ya Chen, Xue-Jiao Hong, Yin-Ping Liu, Hai-Xia Cai, Song Du, Zhi-Chun Gu, Pei-Zhi Ma

**Affiliations:** ^1^Department of Pharmacy, Henan Provincial People's Hospital, People's Hospital of Zhengzhou University, School of Clinical Medicine, Henan University, Zhengzhou, China; ^2^Department of Cardiovascular Medicine, Henan Provincial People's Hospital, People's Hospital of Zhengzhou University, School of Clinical Medicine, Henan University, Zhengzhou, China; ^3^Department of Pharmacy, Ren Ji Hospital, Shanghai Jiao Tong University School of Medicine, Shanghai, China

**Keywords:** atrial fibrillation, elderly, non-vitamin K antagonist oral anticoagulants, drug utility, real-world, risk factors, prediction, machine learning

## Abstract

**Background:**

Atrial fibrillation (AF) is an arrhythmia that is prevalent globally, and its incidence grows exponentially with aging. Non-vitamin K antagonist oral anticoagulants (NOACs) have been developed in recent years, and it challenges the supremacy of warfarin for thromboembolism prophylaxis in AF. Nevertheless, there are limited data specifically evaluating the real-life use of NOACs in elderly patients with AF in China.

**Methods:**

This is a national, multicenter, non-interventional, cross-sectional study that enrolls patients with AF aged 75 years and above from 31 institutions across China. Data were collected using the Hospital Information System. The primary outcomes include (1) profiles of NOAC use in the elderly; (2) frequency of inappropriate NOAC use based on guidelines and approved labeling recommendations; (3) exploring potential risk factors related to NOACs inappropriate use; and (4) creating a prediction tool for inappropriate NOACs use.

**Conclusion:**

The results of this study reveal the prevalence, risk factors, and corresponding prediction tool of inappropriate NOACs use in older patients with AF in China, as well as provide valuable insights into the clinical application of NOACs in high-risk populations in the real-world setting.

**Clinical trial registration:**

www.ClinicalTrials.gov, identifier: NCT 05361889.

## Introduction

Atrial fibrillation (AF) is an arrhythmia that is prevalent globally, and its incidence grows exponentially with aging (1). AF increases the risk of stroke and its sequelae. It also leads to severe complications, such as impaired quality of life, heart failure, dementia, disability, and even death. More frequent hospitalization and treatment of these complications substantially increase the economic burden of the disease (2–5).

Oral anticoagulants (OACs) are the cornerstone of stroke and/or systemic embolism (SE) prevention for patients with non-valvular atrial fibrillation (NVAF) (6, 7). Although dose-adjusted warfarin has been the primary treatment for oral anticoagulation for decades, non-vitamin K antagonist oral anticoagulants (NOACs) have been developed more recently and have challenged the supremacy of warfarin for thromboembolism prophylaxis in NVAF (8–11). NOACs comprise dabigatran (factor IIa inhibitor), rivaroxaban, apixaban, and edoxaban (factor Xa inhibitors), and each of these agents has been proven to be equivalent to or even better than warfarin in terms of effectiveness and safety for stroke prevention in NVAF (12). Current guidelines recommend NOACs as the first-line OAC agents, including for the older adult population (6, 7, 12, 13).

Although advanced chronological age by itself is not a contraindication for anticoagulation in AF, in the real clinical context, the appropriate use of NOAC prescriptions is still challenging for several reasons. First, on the subjective side, many clinicians are hesitant to comply with approved NOAC dosing recommendations for older adults due to concerns posed by frailty, liver or kidney function impairment, polypharmacy, complex comorbidities, prior falls, contraindications, and history of bleeding or potential bleeding risk (14). Second, dosages may be varied to account for ethnic factors. For instance, Asian patients with AF tend to be leaner and shorter and have a greater bleeding risk. This population often receives off-label dose-reduced NOAC more frequently than in Western countries (15). Third, approval was granted by the NOACs' National Medical Products Administration (NMPA) at specific doses as adjusted for age, renal function, body weight, or concomitant administration of other drugs. Therefore, the optimal use of NOACs depends on a thorough understanding of drug labels and the knowledge of definitive guidelines and consensus opinions (16). Finally, some hospitals do not have reliable access to important NOAC formulations (17), precluding their rational use. In previous studies, such inappropriate use of NOACs was problematic because it is associated with adverse events and harmful clinical outcomes, including an increased risk of stroke, thromboembolism, cardiovascular hospitalizations, bleeding, and even all-cause mortality (18, 19).

Since most available data on NOAC practices have been established within the framework of a cardiology setting and in the general population, data on Chinese-specific real-life NOACs use, such as in older adults, are underrepresented. Of note, older Chinese individuals with AF account for a high percentage of the total number of patients with AF. This population is considered to be more susceptible to bleeding. The net clinical benefit of vitamin K antagonist (VKA) use in older adults with AF is barely satisfactory (20). Therefore, collecting data on case mix, clinical characteristics, and treatment for older adults with AF is valuable for improving the management of OAC use in real-world practice. In addition, some studies explored risk factors that influence the appropriateness of NOACs. However, they did not establish the related prediction model (21, 22). Therefore, we will conduct a study to (a) investigate profiles of NOAC use in older individuals, (b) determine the rate of inappropriate NOAC use, (c) explore potential risk factors related to inappropriate NOAC use, and (d) conduct a model for inappropriate use of NOACs.

## Materials and methods

### Data sources

**Figure 1:**
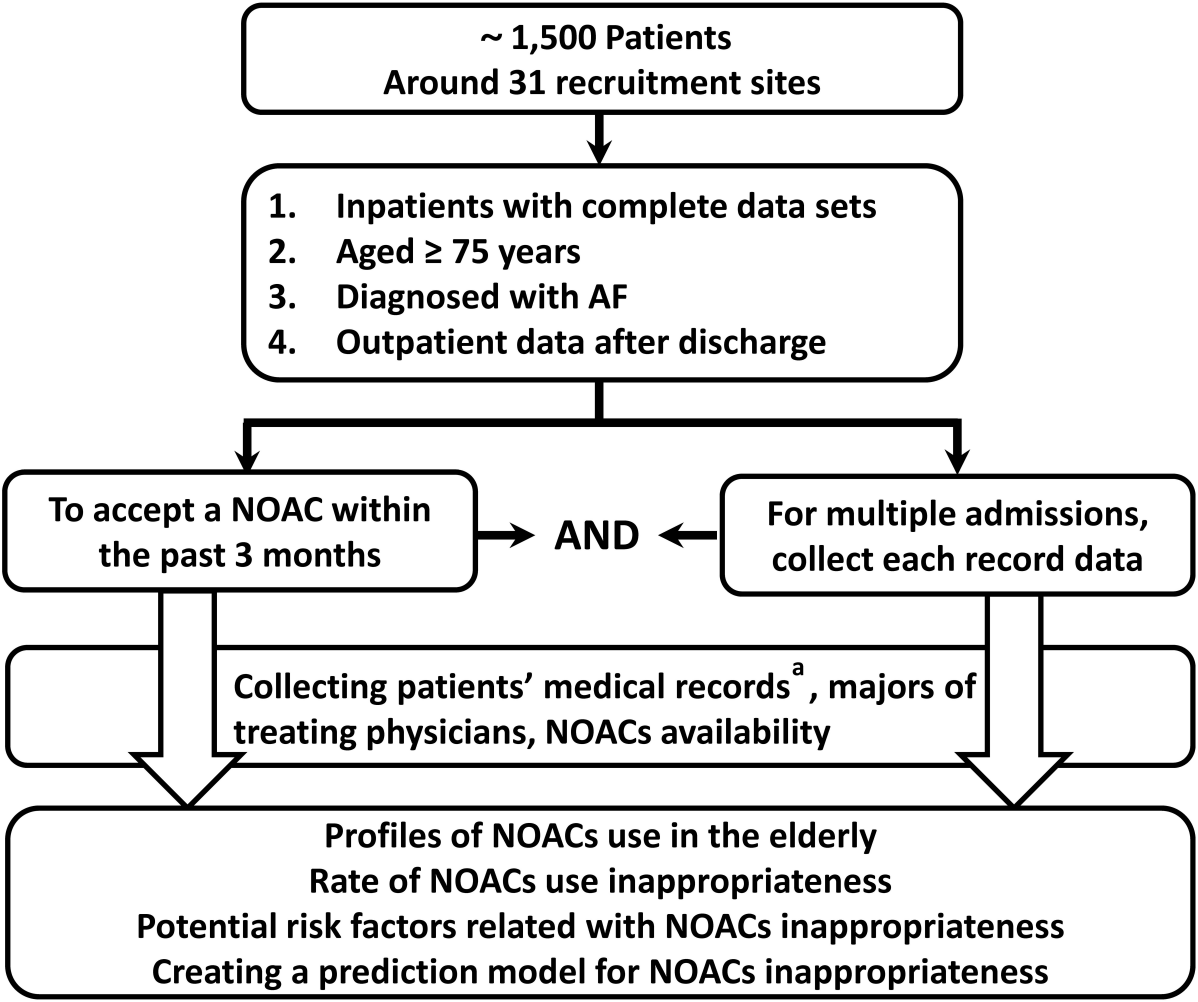
Design schematic of this registry. NOAC, non-vitamin K antagonist oral anticoagulant. a: Including the following details: basic information, disease information, history, latest laboratory data, co-administrations with NOACs, risk factors for thromboembolism (CHA_2_DS_2_-VASc score), and risk factors for bleeding (HAS-BLED score).

This study is proposed as a national, multicenter, non-interventional, cross-sectional study. Individual patient data will be obtained from the ReAl-life multIceNter outcomes registry for Better antithrOmbotic strategies in patients With AF (RAINBOW-AF), an ongoing national AF registration conducted at 31 secondary- or tertiary-care hospitals in China. The purpose of the RAINBOW-AF registry is to collect medical records data of contemporary patients with AF and to determine the relationship between comprehensive assessment and better anticoagulation outcomes in patients with AF. In this study, we will include older adults (aged ≥75 years) who will be receiving a NOAC for NVAF. Considering that this population is regarded as high-risk in the stroke risk-stratification tool (CHA_2_DS_2_-VASc score of at least 2) for NVAF, all selected patients will have strong indications for anticoagulation. The study initiative will be performed based on the Hospital Information System (HIS) (Figure 1). As part of the assessment, this study will be applied for the expansion and spread of a quality promotion initiative for a better NOAC strategy.

### Site selection

The study will be carried out using data given by inpatients from 12 provinces, two municipalities, and one autonomous region across mainland China, with a focus on regions and medical staff features. Suitable site enrollment will be adopted to guarantee geographic heterogeneity and diversity across practice categories (e.g., secondary graded and tertiary hospitals; teaching and general hospitals) and prescriber type (e.g., general practitioner, neurologist, cardiologist, electrophysiologist, and other specialists). In this way, we expect to register a representative sample of patients with NVAF in mainland China; this will limit any bias introduced by the selection of patient criteria, physician specialty, or extent of experience. Around 31 recruitment sites will be activated to select participants for the study.

### Patient recruitment criteria

Eligibility criteria include the following: inpatients with complete data sets; age ≥75 years; diagnosis of AF confirmed by electrocardiogram; use of Holter monitor, pacemaker, implantable device or clinic note, or hospital record of such intervention; or documentation of NOAC therapy for AF regardless of prescriber within the past 3 months. For multiple related admissions, each admission data will be recorded to avoid an omission. To minimize the deviation in the selection of the population and to obtain more sufficient information, the outpatient follow-up data after discharge will also be documented using HIS (Table 1).

**Table 1:** Inclusion and exclusion criteria.

**Inclusion criteria**	**Exclusion criteria**
• Inpatients with complete data sets. To minimize the deviation in the selection of the population and obtain more sufficient information, the outpatient follow-up data after discharge will also be documented *via* Hospital Information System	• AF resulting from reversible causative factors (e.g., thyroid disease, postoperative AF, pulmonary embolism)
• Aged ≥ 75 years	• Have additional indication for anticoagulation treatment apart from AF (e.g., venous thromboembolism, hip/knee replacement surgery, left atrial/ventricular thrombus)
• Diagnosed with AF (e.g., by electrocardiogram, Holter monitor, pacemaker, implantable device, or a history of these interventions in any clinic note or hospital record)	• Bleeding history in critical organs (e.g., intracranial, intraocular, or gastrointestinal bleeding)
• To accept a prescription of NOAC therapy for AF whoever the prescriber within the past 3 months (with the rationale that such patients may have exceptional circumstances preventing long-term anticoagulation or lack of an appropriate indication for long-term anticoagulation)	• Current participation in an ongoing clinical trial of NOAC anticoagulation for AF
• For multiple related admissions, each admission data will be recorded to avoid an omission. For example, patients with variable bleeding factors that could be a relative contraindication or an absolute contraindication that could still be inappropriately used	• Illogical data, missing or insufficient data

*AF, atrial fibrillation; NOAC, non-vitamin K antagonist oral anticoagulant*.

Exclusion criteria include NVAF due to reversible causes (e.g., thyroid disease, postoperative AF, and pulmonary embolism), having an additional indication for anticoagulation treatment apart from AF (e.g., venous thromboembolism and hip/knee replacement surgery), bleeding history in critical organs, current participation in an ongoing clinical trial of NOAC anticoagulation for AF, and incomplete information (illogical data and missing or insufficient data); any patients with such history will also be excluded.

### Ethics and informed consent

The study will be organized and coordinated by the Henan Provincial People's Hospital. The study protocol complies with Good Clinical Practice standards for drugs and the ethical guidelines specified in the revised Declaration of Helsinki (2013). The Henan Provincial People's Hospital Institutional Review Board has approved the RAINBOW-AF registry (approval number: 2022-0406), and the trial was registered at ClinicalTrials.gov (NCT 05361889). Data extracted from medical records will be de-identified and anonymized before analysis; therefore, informed consent is waived for this study. This study will also follow the STROBE reporting checklist and the Transparent Reporting of a Multivariable Prediction Model for Individual Prognosis or Diagnosis (TRIPOD) reporting guidelines.

### Data collection

Data will be entered into a web-based case report form, enabled with dynamic data reviews for patient features, expected data ranges, and mandatory fields. It will be derived primarily from three different sources: patients' electronic medical records (including demographics, clinical characteristics, medical management, laboratory measurements, imaging parameters, and drug information), the grade level of treating physicians, referring to the clinical expertise or academic background of clinicians, and the availability of NOACs at each hospital. The specific details include participants' sex, age, body weight, lifestyle, comorbidities (such as diabetes, coronary heart disease, and heart failure), history, latest laboratory data, and risk factors for thromboembolism and bleeding. In addition, co-administration of drugs (such as antiplatelet agents and antiarrhythmic therapy), appearances after NOAC treatment (incidence of inappropriate use), the expertise of physicians, and NOAC availability from that institution will also be documented (Table 2).

**Table 2:** Details of data collection.

**Data collection**	**Interpretation**
• Basic information	Participants' sex, age, body weight, marital status, lifestyle (current smoking and drinking status), educational status, place of residence (rural or urban)
• Disease Information	Type of AF, comorbidities (correlate with stroke and bleeding risk, e.g., anemia, MI, PAD), procedures or surgical history (PCI, CABG, or RFCA)
• History	History of thromboembolism and related hemorrhagic events (major bleeding is defined based upon ISTH criteria and incidences apart from major bleeding are considered as non-major bleeding), fall history
• Latest laboratory data	Serum creatinine levels, hemoglobin, bilirubin, liver function (Child-Pugh score), and renal function (CrCl, calculated using Cockroft-Gault formula)
• Co-administration with NOACs	NOAC medication type, antiplatelet agent, interacting combination medications with NOACs, such as antiarrhythmic therapy, itraconazole, ketoconazole, ritonavir, etc.
• Risk factors for thromboembolism (CHA_2_DS_2_-VASc score)	CHA_2_DS_2_-VASc score is a rating of risk for stroke in patients with AF, items of 1 point each for congestive heart failure, hypertension, diabetes mellitus, vascular disease, Age 65–74 years, sex category [female], and 2 points each for a history of a stroke, TIA, or age ≥ 75 years)
• Risk factors for bleeding (HAS-BLED score)	HAS-BLED score is a rating of risk for bleeding in patients with AF, 1 point each for hypertension, abnormal renal/liver function, stroke, bleeding history or predisposition, labile INR, age 65 years or greater, drugs/alcohol concomitantly
• Expertise of prescriber	Cardiologist or not
• NOACs availability from that institution	Some institutions do not have access to certain NOACs, which can influence the NOAC inappropriateness

*AF, atrial fibrillation; MI, myocardial infarction; PAD, peripheral arterial disease; PCI, percutaneous coronary intervention; CABG, coronary-artery bypass grafting; RFCA, radiofrequency catheter ablation; ISTH, International Society of Thrombosis and Hemostasis; CrCl, creatinine clearance rate; TIA, transient ischemic attack; INR, international normalized ratio; NOAC, non-vitamin K antagonist oral anticoagulant*.

### Evaluation of the appropriate NOACs use

**Figure 2:**
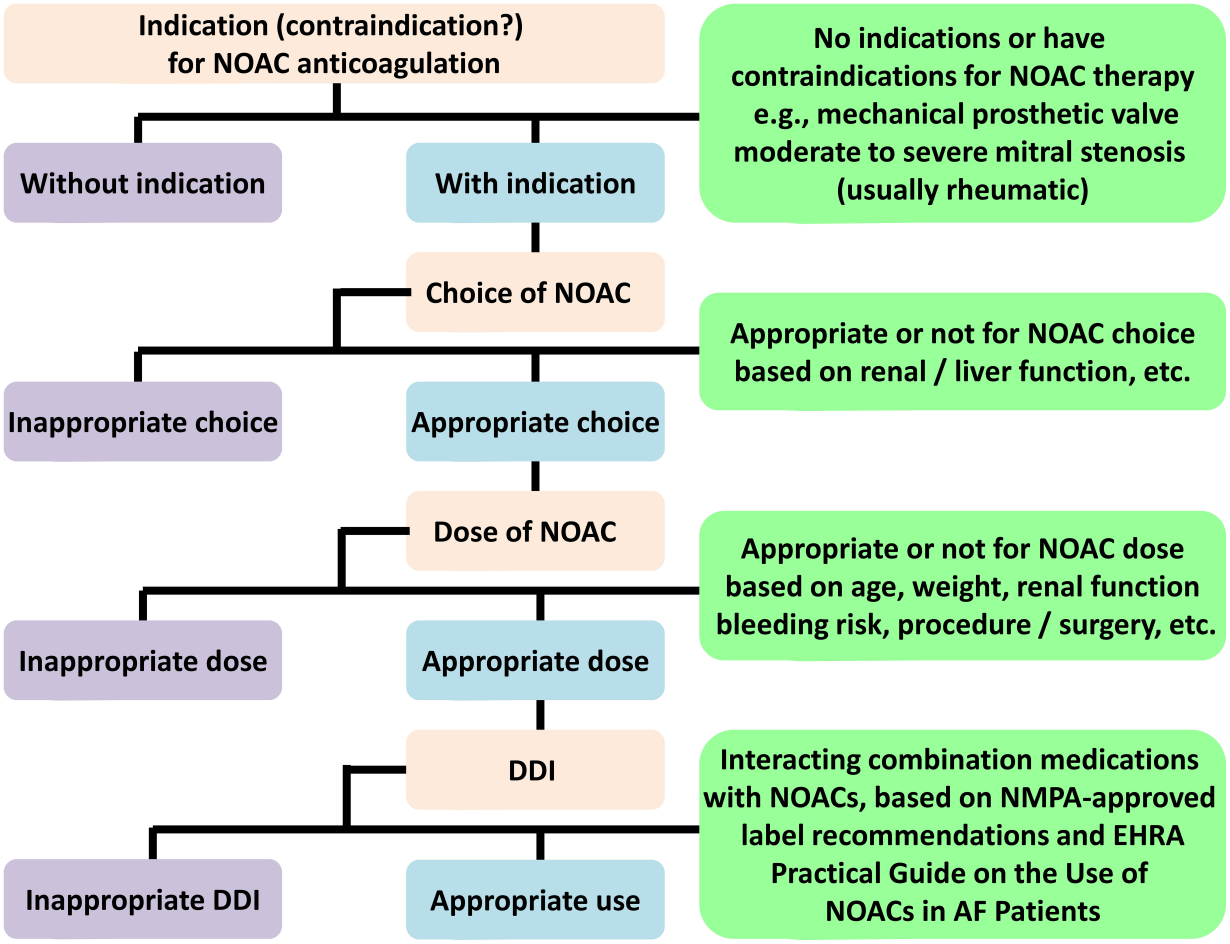
NOACs evaluation flowchart. NOAC, non-vitamin K antagonist oral anticoagulant; DDI, drug-drug interaction; NMPA, National Medical Products Administration; EHRA, European Heart Rhythm Association; AF, atrial fibrillation.

Different nations do not always adopt the standard indications for NOAC prescription. Local policies, such as formulary committees, regulatory approval, and cost-effectiveness, all influence NOAC labeling recommendations (23). In this study, we will adopt an adaptive design to account for a summary of product characteristics, and analyze prevalent NOAC strategies based on indications, NOAC selection, or dosages (Figure 2). We use the recommendations given by NMPA for each agent and the 2021 European Heart Rhythm Association (EHRA) practical guide on the use of NOACs for patients with AF (24). Patients for whom the selected NOAC was contraindicated can be categorized as inappropriate use. If there exist no absolute contraindications, the protocol can evaluate appropriateness according to patient-specific features, like age, weight, renal function, Child-Pugh classification, specific drug–drug interactions, hemoglobin level, and bleeding risk. In cases of discrepancy between the EHRA recommendations and the NMPA recommendations, we adopt the NMPA-approved label recommendations as the standard for determining the appropriateness (Table 3). Patients will be classified as either (a) NOAC-appropriate (rational use of NOAC according to the standardized criteria) or (b) NOAC-inappropriate (irrational use of NOAC according to the standardized criteria).

**Table 3:** Approved dosing regimens for the NOACs package inserts for NVAF in the Mainland China.

**Medication**	**Regulations from the NMPA (Mainland China)**
	NMPA (Mainland China) (Revised: 06/2020)
Dabigatran	• Full dose: 150 mg twice daily
	• 110 mg twice daily, if: -age ≥ 80 years -concomitant verapamil
	• Daily dose of 300 mg or 220 mg according to an individual evaluation of the thromboembolic risk and bleeding risk: -age 75–80 years -moderate renal impairment (CrCl 30–50 mL/min) -gastritis, esophagitis, or gastroesophageal reflux -other increased bleeding risk ^a^
Rivaroxaban	NMPA (Mainland China) (Revised: 07/2020)
	• Full dose: 20 mg once daily with food when CrCl ≥ 50 mL/min
	• 15 mg once daily with food, if: -CrCl 15 – 49 mL/min
Apixaban	NMPA (Mainland China) (Revised: 02/2019)
	• Not approved
Edoxaban	NMPA (Mainland China) (Revised: 07/2021)
	• Full dose: 60 mg once daily
	• 30 mg once daily with one or more of the following clinical factors: -CrCl 15 – 50 mL/min -body weight ≤ 60 kg -concomitant use of the following P-gp inhibitors: ciclosporin, erythromycin, dronedarone, or ketoconazole

NVAF, non-valvular atrial fibrillation; NOAC, non-vitamin K antagonist oral anticoagulants; NMPA, National Medical Products Administration; P-gp, P-glycoprotein; CrCl, creatinine clearance rate.

a : Other increased bleeding risks include: Strong P-gp inhibitors; mild to moderate P-gp inhibitor co-medication (e.g., quinidine, verapamil, ticagrelor, and amiodarone); low body weight (<50 kg); acetylsalicylic acid (ASA) and other platelet aggregation inhibitors, e.g., clopidogrel; selective serotonin norepinephrine re-uptake inhibitors (SNRIs), selective serotonin re-uptake inhibitors (SSRIs), and non-steroidal anti-inflammatory drugs (NSAID); other medicinal products that may impair hemostasis; functional platelet defects or thrombocytopenia; major trauma and recent biopsy; and bacterial endocarditis.

Since apixaban has not been fully studied in Chinese populations, the NMPA did not approve the NVAF indication for apixaban; thereby, we have excluded it from this study. Rivaroxaban, given 15 mg daily (or 10 mg, based on creatinine clearance rate [CrCl]), in combination with clopidogrel, was also used in patients with AF and recent percutaneous coronary intervention (PCI) or acute coronary syndromes (ACS) in the PIONEER AF-PCI trial. Therefore, we considered that both doses are appropriate for such patients in our study. The NMPA recommends a daily dose of 220 mg for dabigatran, which can be given to patients with CrCl 30–50 ml/min, aged ≥75 years, or with increased bleeding risk. The NOAC prescription was deemed reasonable for patients aged ≥75 years.

### Quality control

Standard criteria for NOAC appropriateness were drafted by the two highly experienced pharmacists (S-JZ and B-YC) after obtaining consent from all collaborators. A steering committee, comprising of an independent panel of experts (organized by Z-CG) blinded to RAINBOW-AF data independently checked the entire algorithm program (including recommendation-consistency, under-dosing, or over-dosing) to validate its results. A standard electronic data capture form was devised to accumulate data. To explain each data element as clearly as possible, consensus must be met prior to the entry of study data to discuss the specifics. RAINBOW-AF health personnel who are involved in the care of the study participants will receive rigorous training with periodical quality control inspection prior to the study. All study outcomes are defined based on the diagnosis at the first discharge to avoid misclassification. Manual chart review results must conform to the program of the algorithm revealed above in all cases. Data monitoring will be implemented by the coordinating administrators to determine the integrity and accuracy of data input.

### Primary outcomes

The primary outcomes include (a) profiles of NOAC use in the elderly; (b) the rate of inappropriate NOAC use according to the guidelines and labeling recommendations; (c) potential risk factors related to inappropriate NOAC use; and (d) creation of a prediction model for inappropriate NOAC use. First, we will try to identify the characteristics of participants who were given appropriate NOAC agents and those who were not from a number of several variables (e.g., demographics, clinical, management, expertise of treating clinicians, NOAC availability, and the prevalence of potentially inappropriate NOAC use). Next, using this binary outcome, a logistic model will be fitted to quantify risk factors correlated with these variables with selections of NOAC-inappropriate regimens. Lastly, we will create a prediction model using both logistics and machine learning method for predicting inappropriateness in the entire cohort.

### Sample size calculation

This study is designed as a multicenter cross-sectional study. Assuming that each institution can treat 300–500 patients with AF per year, patients over 75 years old account for 20–30% and the rate of NOAC use is ~50–70%, with a 2% error range and 95% confidence interval (CI). The drop-out rate is not considered in the calculation as the primary outcomes will be evaluated during hospitalization; the drop-out rate is expected to be very low. To get a representative result, we calculated that a sample size of 1,500 patients would be sufficient. Since this is an event-driven study (the rate of inappropriate NOAC use), the total number of patients may change as necessary according to the cumulative number of target events.

### Model development process

#### Predictors and outcomes

Based on demographics, clinical, medical management, patient characteristics, CHA_2_DS_2_-VASc score, HAS-BLED score, the expertise of treating physicians, NOAC availability, and clinical relevance, we can identify the variables potentially related to inappropriate NOAC use. Variables above will be extracted from the medical records in each participating institution using an *a priori* designed form. The target outcome will be NOAC inappropriateness. Patients with more than 30% missing data will be excluded. For patients with partially missing data, the Random Forest (RF) algorithm will be used for data-level computation.

#### Data separation and feature filtering

In this process, the data will be randomly divided into training and test sets in a ratio of 8:2, which will be used for the model establishment and verification, respectively. Feature selection will be performed using the Sequence Forward Selection algorithm based on RF. For each group, the algorithm will search from the empty set and subsequently add one variable to the feature subset each time to achieve an optimal performance, which is measured by the F1 score.

#### Machine learning model establishment, evaluation, and interpretation

Both linear and non-linear machine learning models will be applied to the sets, which include logistic regression, RF, support vector machine, gradient boosting tree (such as XGBoost, LightGBM, Adaboost, and Catboost), and attentive interpretable tabular learning (TabNet). The prediction performance of all models will be evaluated through five measures (area under the receiver operating characteristics [AUROC] curve, precision, recall, F1 score, and accuracy). The acceptably performing machine learning models will be selected according to AUROC. Next, we will calculate the importance scores of the features using the above-chosen algorithms. Features with higher importance scores are more closely related to the accurate prediction of NOAC inappropriate use. Finally, features ranked in the top 50% based on normalized importance scores in selected models will be determined as major predictors and depicted in radar plots.

### Statistical analyses

Categorical data will be presented as frequencies and be compared by Chi-squared or Fisher's exact test as appropriate. Continuous data will be reported as mean ± standard deviation or median and interquartile range (IQR) and analyzed by either Student's *t*-test or the Mann–Whitney *U* test. In this study, data will be extracted and summarized using Excel 2016. Univariate and multivariate logistic regression analyses will be applied to ascertain the correlation between candidate variables and NOAC inappropriate use. Multicollinearity between the variables will be shifted based on the variance inflation factor (VIF; VIF > 5 is considered strong collinearity) (25). Two criteria will be considered necessary for a variable to be incorporated into the final prediction model: (a) a univariate *p-*value indicative of NOAC prescription inappropriateness ≤0.05 and (b) a plausible connection with risk factors from NOAC inappropriate prescriptions based on previously published research. The risk tendency of inappropriate NOAC use among risk stratifications will be evaluated by the Cochran-Armitage trend test (26). Machine learning algorithms will be built based on the Scikit-learn package (version 0.22.2). Interaction analyses will be used to compare predictive performance between machine learning models. Statistics will be performed employing STATA software (version 12.0, Stata Corporation LLC, College Station, United States), and a *p* < 0.05 will be considered to be statistically significant.

## Discussion

This is a national, multicenter, non-interventional, cross-sectional study driven by pharmacists to explore NOAC use appropriateness in older adults with AF in the real-world setting, which includes the prevalence of potentially inappropriate NOAC use and relevant influencing factors, and to derive a clinically practical prediction model for predicting the risk of inappropriate NOAC use based on explored risk factors. The prediction model will be applied in routine clinical practice to identify patients potentially at risk due to inappropriate NOAC use and to optimize anticoagulation management for older adults with AF.

The research data will come from the RAINBOW-AF registry, which will function as a post-marketing surveillance study after the transition from a single available traditional OAC (warfarin) to the target-specific NOAC agents. The RAINBOW-AF registry enables aggregation and integration of information on OAC use, safety, and effectiveness from 31 medical institutions in 7 different regions of China (East, South, Central, North, Northwest, and Northeast China and Southwest Asia). From the cohort perspective, baseline characteristics, contemporary anticoagulant management practices, and treatment outcomes will be described in the setting of real clinical scenarios in Chinese patients with AF. A remarkable feature of the RAINBOW-AF register is that it includes patients from all levels of medical institutions in mainland China, including those from secondary-graded hospitals or general departments. Thus, patients can be well-represented at all levels in mainland China, regardless of anticoagulant strategies. We plan to include patients who have been discharged after only a short stay in the hospital and their outpatient follow-up information because these groups of patients also frequently take NOACs.

High-quality anticoagulant treatment is crucial in guaranteeing the effectiveness and safety of OAC administration in AF patients. The benefits of OACs in NVAF may be impossible to achieve if anticoagulant regimens are prescribed inappropriately (27). As NOAC use has become more pervasive, off-label prescribing has become a global issue. Several studies have outlined the adverse clinical consequences of off-label prescribing. The ENGAGE AF-TIMI 48 trial illustrated the outcome of under-dosing of edoxaban (11). The edoxaban 30/15 mg group posed a significantly higher ischemic stroke risk when compared to the well-controlled vitamin K antagonist group; this resulted in the disapproval of this dosing regimen for clinical application (11). In the ORBIT-AF II registry, off-label doses of NOACs presented an increased risk for adverse events than the recommended NOAC dosage protocols (27). In particular, NOAC over-dosing was associated with increased all-cause mortality, while NOAC under-dosing was associated with higher rates of hospitalization for cardiovascular conditions (27). Another study demonstrated that inappropriate NOAC prescriptions were more likely to occur in the older adult population (28). This is the most worrisome finding because the risk of stroke increases significantly with age. Inappropriate dosage exposes high-risk patients to potential hazards of disabling or even fatal strokes.

Previous studies report relevant risk factors for inappropriate NOACs use. In the SAGE-AF (Systematic Assessment of Geriatric Elements in Atrial Fibrillation) cohort, a potentially inappropriate NOAC dose was prescribed to nearly a quarter of older adults. This stemmed from patients being older, having poor renal function, and higher CHA_2_DS_2_-VASc scores (16). Another Korean study found that NOAC label adherence was approximately 60%, and the risk factors of NOAC under-dosing were independently related to old age (≥75 years), female sex, lower body weight (< 60 kg), renal impairment (CrCl < 50 ml/min), hypertension, previous stroke/transient ischemic attack (TIA)/thromboembolic events, bleeding history, and concomitant dronedarone or antiplatelet agent use (29). In the ORBIT-AF II registry (18), compared with those whose NOAC dose was appropriately reduced, patients accepting inappropriate dose reductions were younger and had lower bleeding risk scores. Physician assessment may have a strong impact on inappropriate NOAC dosing; however, there may also be drug-specific factors. In Mainland China, information on practice patterns of NOAC administration and the use of NOACs for licensed indications in older patients remains scarce.

Currently, few studies have focused attention on the prediction model of the risk factors of NOAC inappropriateness in elderly patients with AF. Considering that inappropriate NOACs doses are prescribed to a fair percentage of older patients with AF, with most being under-dosed, the development of a practical prediction model with reliable predictability is of great value. To our knowledge, this will be the first study to establish a feasible prediction model for predicting inappropriate NOAC use in older Chinese patients with AF. In the prediction model of probabilistic estimation, we will combine the above independent predictors and the risk factor variables from previously published research. Accordingly, we believe that the prediction model could be applied as a simpler and more effective tool for clinical decision-making for elderly patients with AF.

### Strengths and limitations

The main strengths of this study are believed to be as follows: First, we will include data from a national, comprehensive, and diversified cohort of older patients with AF and will perform in-depth phenotyping of NOAC use to help refine best practices for older adults with AF. Second, most studies evaluating the appropriateness of NOAC primarily focus on dosage in the general population, while our study will evaluate the rationale of NOAC decision-making for older adults from multiple perspectives: patient factor (patients' demographics and categories of treating physicians) and institution layer (the availability of certain NOACs in that center). Additionally, this study will build the first prediction model to predict the inappropriateness of NOAC in older Chinese patients with AF.

Inevitably, this study has some inherent limitations. First, due to the inherent restriction placed by its design, this study will not evaluate the outcomes of older adults with AF treated with inappropriate anticoagulation therapy, nor will there be a follow-up for efficacy evaluation. Nevertheless, its primary purpose is to establish a reliable prediction model in older adults with NVAF receiving NOAC. Second, data will be dependent on the quality of medical record extraction, so that residual and unmeasured covariates among the associated variables may influence the results, even after strict measurement and recording of crucial variables and avoidance of confounding factors. Last, the prediction model needs independent validation in other cohorts to establish its utility for clinical use, not least in older outpatients taking NOACs.

## Conclusion

This study will provide unique and valuable data on the feasibility of NOAC use in older adults with AF. This will be the first study to establish a prediction model to predict inappropriate NOAC use in this high-risk population.

## Ethics statement

The studies involving human participants were reviewed and approved by Henan Provincial People's Hospital Institutional Review Board. Written informed consent for participation was not required for this study in accordance with the national legislation and the institutional requirements.

## Author contributions

P-ZM and Z-CG are the guarantors of the entire manuscript. S-JZ and Z-CG participated in the study conception and design, drafting, and critical revision. S-JZ and B-YC contributed to the project administration. All authors contributed to the acquisition, analysis, and interpretation of data, and they agreed to be held accountable for all aspects of the work and approved the submitted version.

## Funding

This study was supported by the Research Project Henan Provincial Department of Science and Technology (No.: 212102310656) and the Joint Construction Project of Henan Medical Science and Technology Program (No.: LHGJ20210052).

## Conflict of interest

The authors declare that the research was conducted in the absence of any commercial or financial relationships that could be construed as a potential conflict of interest.

## Publisher's note

All claims expressed in this article are solely those of the authors and do not necessarily represent those of their affiliated organizations, or those of the publisher, the editors and the reviewers. Any product that may be evaluated in this article, or claim that may be made by its manufacturer, is not guaranteed or endorsed by the publisher.

## References

[1] Zhao S Hong X Cai H Liu M Li B Ma P. Antithrombotic management for atrial fibrillation patients undergoing percutaneous coronary intervention or with acute coronary syndrome: an evidence-based update. Front Cardiovasc Med. (2021) 8:660986. 10.3389/fcvm.2021.660986 34262952 PMC8273244

[2] Bencivenga L Komici K Nocella P Grieco FV Spezzano A Puzone B . Atrial fibrillation in the elderly: a risk factor beyond stroke. Ageing Res Rev. (2020) 61:101092. 10.1016/j.arr.2020.101092 32479927

[3] Alrumayh A Alobaida M. Catheter ablation superiority over the pharmacological treatments in atrial fibrillation: a dedicated review. Ann Med. (2021) 53:551–57. 10.1080/07853890.2021.1905873 33783271 PMC8018546

[4] Sussman M Di Fusco M Tao CY Guo JD Gillespie JA Ferri M . The IMPact of untReated nOn-Valvular atrial fibrillation on short-tErm clinical and economic outcomes in the US Medicare population: the IMPROVE-AF model. J Med Econ. (2021) 24:1070–82. 10.1080/13696998.2021.1970954 34415229

[5] Goulart AC Olmos RD Santos IS Tunes G Alencar AP Thomas N . The impact of atrial fibrillation and long-term oral anticoagulant use on all-cause and cardiovascular mortality: a 12-year evaluation of the prospective Brazilian Study of Stroke Mortality and Morbidity. Int J Stroke. (2021) 17:48–58. 10.1177/1747493021995592 33527882

[6] Hindricks G Potpara T Dagres N Arbelo E Bax JJ Carina B-L . 2020 ESC Guidelines for the diagnosis management of atrial fibrillation developed in collaboratio n with the European Association for Cardio-Thoracic Surgery (EACTS): The Task Force for the diagnos is management of atrial fibrillation of the European Society of Cardiology (ESC) Developed with the special contribution of the European Heart Rhythm Association (EHRA) of the ESC. Eur Heart J. (2021) 42:373–498. 10.1093/eurheartj/ehaa612 32860505

[7] Heidenreich PA Estes NAM Fonarow GC Jurgens CY Kittleson MM Marine JE . 2020 Update to the 2016 ACC/AHA clinical performance and quality? Measures for adults with atrial fibr illation or atrial flutter: a report of the American College of Cardiology/American Heart Associatio n Task Force on performance measures. J Am Coll Cardiol. (2021) 77:326–41. 10.1016/j.jacc.2020.08.037 33303319

[8] Connolly SJ Ezekowitz MD Yusuf S Eikelboom J Oldgren J Parekh A . Dabigatran versus warfarin in patients with atrial fibrillation. N Engl J Med. (2009) 361:1139–51. 10.1056/NEJMoa0905561 19717844

[9] Patel MR Mahaffey KW Garg J Pan G Singer DE Hacke W . Rivaroxaban versus warfarin in nonvalvular atrial fibrillation. N Engl J Med. (2011) 365:883–91. 10.1056/NEJMoa1009638 21830957

[10] Granger CB Alexander JH McMurray JJ Lopes RD Hylek EM Hanna M . Apixaban versus warfarin in patients with atrial fibrillation. N Engl J Med. (2011) 365:981–92. 10.1056/NEJMoa1107039 21870978

[11] Giugliano RP Ruff CT Braunwald E Murphy SA Wiviott SD Halperin JL . Edoxaban versus warfarin in patients with atrial fibrillation. N Engl J Med. (2013) 369:2093–104. 10.1056/NEJMoa1310907 24251359

[12] Andrade JG Aguilar M Atzema C Bell A Cairns JA Cheung CC . The 2020 Canadian Cardiovascular Society/Canadian Heart Rhythm Society Comprehensive Guidelines for the management of atrial fibrillation. Can J Cardiol. (2020) 36:1847–948. 10.1016/j.cjca.2020.09.001 33191198

[13] Liao J-N Chan Y-H Kuo L Tsai C-T Lim S-S Chao T-F. Optimal anticoagulation in elderly patients with atrial fibrillation: which drug at which dose? Kardiol Pol. (2022) 80:128–36. 10.33963/KP.a2022.0046 35167115

[14] Bahri O Roca F Lechani T Druesne L Jouanny P Serot J-M . Underuse of oral anticoagulation for individuals with atrial fibrillation in a nursing home setting in france: comparisons of resident characteristics and physician attitude. J Am Geriatr Soc. (2015) 63:71–6. 10.1111/jgs.13200 25597559

[15] Shen N-N Zhang C Hang Y Li Z Kong L-C Wang N . Real-world prevalence of direct oral anticoagulant off-label doses in atrial fibrillation: an epidemiological meta-analysis. Front Pharmacol. (2021) 12:581293. 10.3389/fphar.2021.581293 34122056 PMC8188240

[16] Sanghai S Wong C Wang Z Clive P Tran W Waring M . Rates of potentially inappropriate dosing of direct-acting oral anticoagulants and associations with geriatric conditions among older patients with atrial fibrillation: the SAGE-AF study. J Am Heart Assoc. (2020) 9:e014108. 10.1161/JAHA.119.014108 32146898 PMC7335533

[17] Ding Z Zhang C Qian YY Wang N Gu ZC Xu H . Rationale and design of a prospective, multicenter, cross-sectional study of appropriateness evaluati on of the prescription of non-vitamin K antagonist oral anticoagulants for Chinese atrial fibrillati on patients (Chi-NOACs-AF trial). Ann Transl Med. (2021) 9:580. 10.21037/atm-20-6893 33987278 PMC8105835

[18] Steinberg BA Shrader P Pieper K Thomas L Allen LA Ansell J . Frequency and outcomes of reduced dose non–vitamin k antagonist anticoagulants: results from ORBIT-AF II (the outcomes registry for better informed treatment of atrial fibrillation II). J Am Heart Assoc. (2018) 7:e007633. 10.1161/JAHA.117.007633 29453305 PMC5850192

[19] Ono T Ikemura N Kimura T Ueda I Tanaka H Tokuda H . Contemporary trend of reduced-dose non-vitamin K anticoagulants in Japanese patients with atrial fibrillation: a cross-sectional analysis of a multicenter outpatient registry. J Cardiol. (2019) 73:14–21. 10.1016/j.jjcc.2018.09.003 30487057

[20] Patti G Pecen L Lucerna M Huber K Rohla M Renda G . Net clinical benefit of non-vitamin k antagonist vs vitamin k antagonist anticoagulants in elderly patients with atrial fibrillation. Am J Med. (2019) 132:749–57.e5. 10.1016/j.amjmed.2018.12.036 30664837

[21] Franchi C Antoniazzi S Proietti M Nobili A Mannucci PM. Appropriateness of oral anticoagulant therapy prescription and its associated factors in hospitalized older people with atrial fibrillation. Br J Clin Pharmacol. (2018) 84:2010–19. 10.1111/bcp.13631 29745441 PMC6089830

[22] Shurrab M Ko DT McElhaney J Henderson M Danon A Quinn KL . Identifying factors that predict the prescription of non–vitamin k antagonist oral anticoagulants in older individuals with atrial fibrillation. J Am Med Dir Assoc. (2019) 20:984–87. 10.1016/j.jamda.2019.01.131 30853427

[23] Zhao S Hong X Cao J Cai H Du S Ma P. Appropriate dosing regimens of non-vitamin k antagonist oral anticoagulants for treatment of patients with non-valvular atrial fibrillation: an evidence-based consideration. Front Pharmacol. (2020) 11:1293. 10.3389/fphar.2020.01293 32973522 PMC7468491

[24] Steffel J Collins R Antz M Cornu P Desteghe L Haeusler KG . 2021 European Heart Rhythm Association Practical Guide on the use of non-vitamin k antagonist oral an ticoagulants in patients with atrial fibrillation. Europace. (2021) 23:1612–76. 10.1093/europace/euab065 33895845 PMC11636576

[25] Vatcheva KP Lee M McCormick JB Rahbar MH. Multicollinearity in regression analyses conducted in epidemiologic studies. Epidemiology. (2016) 6:227. 10.4172/2161-1165.1000227 27274911 PMC4888898

[26] Gu ZC Zhang C Yang Y Wang MG Li HY Zhang GY. Prediction model of in-hospital venous thromboembolism in Chinese adult patients after hernia surgery: the CHAT score. Clin Appl Thromb Hemost. (2021) 27:10760296211051704. 10.1177/10760296211051704 34928746 PMC8725045

[27] Steinberg BA Shrader P Thomas L Ansell J Fonarow GC Gersh BJ . Off-label dosing of non-vitamin k antagonist oral anticoagulants and adverse outcomes. J Am Coll Cardiol. (2016) 68:2597–604. 10.1016/j.jacc.2016.09.966 27978942

[28] Anouassi Z Atallah B Alsoud LO El Nekidy W Al Mahmeed W AlJaabari M . Appropriateness of the direct oral anticoagulants dosing in the Middle East Gulf Region. J Cardiovasc Pharmacol. (2021) 77:182–88. 10.1097/FJC.0000000000000913 32925474

[29] Lee S-R Lee YS Park J-S Cha M-J Kim T-H Park J . Label adherence for non-vitamin k antagonist oral anticoagulants in a prospective cohort of Asian patients with atrial fibrillation. Yonsei Med J. (2019) 60:277. 10.3349/ymj.2019.60.3.277 30799590 PMC6391519

